# Metabolic Syndrome: a challenging health Issue in highly urbanized Union Territory of north India

**DOI:** 10.1186/1758-5996-2-19

**Published:** 2010-03-23

**Authors:** Chetna Mangat, NK Goel, Dinesh K Walia, Neeraj Agarwal, Munesh K Sharma, Jasbinder Kaur, Ram Singh, Gagandeep Singh

**Affiliations:** 1 Department of Community Medicine, Government Medical College Chandigarh, India; 2 Department of Biochemistry, Government Medical College Chandigarh, India; 3 Department of Medicine, Government Medical College Chandigarh, India; 4 Goondiwindi Medical Centre, Goondiwindi, QLD, Australia

## Abstract

**Objectives:**

1. To determine the prevalence of Metabolic Syndrome in adults aged 18 years and above in Chandigarh, India. 2. To determine the socio-demographic factors associated with MS. 3. To determine the agreement between IDF (International Diabetes federation definition) and ATP-III (National Cholesterol Education Program Expert Panel on Detection, Evaluation, and Treatment of High Blood Cholesterol in Adults criteria).

**Methods:**

In a community based cross-sectional study, total 605 subjects aged 18 yrs and above were studied using multistage random sampling.

**Results:**

Prevalence of Metabolic Syndrome was estimated by using IDF and ATP-III criteria. By IDF, Metabolic Syndrome was found in 287 (47.4%) subjects and it was more prevalent among females 171 (59.6%) as compared to males 116 (40.4%). By applying ATP-III overall prevalence was less i.e. 233 (38.5%) but again its prevalence was more among females 141 (44.8%) than males 116 (39.5%). Higher socioeconomic status, sedentary occupation and high body mass index were significantly associated with Metabolic Syndrome.

**Conclusions:**

Metabolic Syndrome is a major health problem in the region and proper emphasis should be given on its prevention and control.

## Introduction

MS is a clustering of abnormalities that confers an increased risk of developing not only cardiovascular disease (CVD) but also type 2 diabetes mellitus [1]. It has reached to epidemic proportions worldwide [2]. About 20-30% of adult population worldwide is suffering from this syndrome [3]. Millions of people in developing countries are facing a double health burden that represents an unsettling modern-day paradox i.e. the impact of poverty-related diseases (associated with infections and nutrition) is being exacerbated by the increasing load of chronic non communicable diseases [4].

MS was firstly defined by World Health Organization (WHO) in 1998 [5], after that many international agencies and organizations purposed various definitions to screen it. Out of these the most widely used definitions are by the National Cholesterol Education Program Expert Panel on Detection, Evaluation, and Treatment of High Blood Cholesterol in Adults (ATP-III) proposed in 2001 [6] and the International Diabetes Federation (IDF) proposed in 2005 [7]. But all the groups agreed on the core components of MS that consist of obesity, insulin resistance, dyslipidemia and hypertension, however the specific factors and their cutoff values used in the various definitions were different to identify MS.

According to a recent systematic review of studies published between 1998 and 2005, has shown the strong association between the MS and the risk of CVD [8]. MS also increases incidence of coronary heart diseases and diabetes [9]. Apart from its association with cardiovascular disorder and diabetes mellitus, it is a common soil for numerous other clinical disorders too and so it has became a matter of great debate.

The prevalence of MS is rapidly increasing in developing countries due to changing lifestyle. Chandigarh is a highly urbanized city (90% of population resides in urban areas) and there seems to be no literature available regarding MS in Chandigarh except a small school based study in adolescents [10]. The present community based study was conducted to screen individuals from rural, urban and slum populations of Chandigarh along with its associated factors.

## Objectives

1. To determine the prevalence of Metabolic Syndrome in adults aged 18 years and above in Chandigarh, India. 2. To determine socio-demographic and some other factors associated with MS. 3. To determine the agreement between IDF and ATP-III.

## Study design

A community based cross-sectional study was conducted in urban and rural and slum populations of Union Territory, Chandigarh, located in northern India.

## Methods

Stratified multistage random sampling was used for selection of study subjects. The whole population was divided in to rural and urban strata. At the first stage, sample of 2 rural and 6 urban wards were selected at random as primary stage units. Within each primary stage unit, a sample of one sector in urban area and one village from rural area were selected at random as secondary stage units. Within selected second stage units a sample of households were surveyed as third stage units. Within selected households, individuals satisfying inclusion criteria were selected to achieve the pre-determined optimum sample size.

In India the prevalence of MS as available in different studies, ranges from 20%-40%. On the basis of pilot study conducted prevalence of 40% was found, by keeping 95% confidence coefficient and allowing 10% permissible error the optimum sample size was found to be 576. Total 606 subjects agreed for biochemical investigations and one blood sample was hemolysed during transportation hence 605 respondents were screened for MS.

Individuals aged 18 yrs and above, irrespective of disease status (diabetics, hypertensives and dyslipidemic patients were not excluded) were screened for MS, only pregnant females were excluded from screening. For the sake of feasibility in terms of time, cost and other considerations, IDF and ATP-III criteria were used for screening of MS [6,7]. Key points regarding these two criteria are given in table 1 and table 2.

**Table 1:** ATP-III definition of Metabolic Syndrome

Three or more of the following five risk factors:	
**Risk factor**	**Defining level**

Central obesity	Waist circumference
• Men	> 102 cm (> 40 in)
• Women	> 88 cm (> 35 in)

Triglycerides	≥ 150 mg/dL (1.7 mmol/L)

HDL cholesterol	
• Men	< 40 mg/dL (1.03 mmol/L)
• Women	< 50 mg/dL (1.29 mmol/L)

Blood pressure	≥ 130/≥ 85 mm Hg

Fasting glucose	≥ 110 mg/dL (6.1 mmol/L)

**Table 2:** IDF definition of Metabolic Syndrome

Central obesity (defined as waist circumference ≥ 90cm for Asian men ≥ 80cm for Asian women, with ethnicity specific values for other groups) plus any two of the following four factors:	
1.	Raised TG level: ≥ 150 mg/dL (1.7 mmol/L), or specific treatment for this lipid abnormality

2.	Reduced HDL cholesterol: < 40 mg/dL (1.03 mmol/L) in males and < 50 mg/dL (1.29 mmol/L) in females, or specific treatment for this lipid abnormality

3.	Raised blood pressure: systolic BP ≥ 130 or diastolic BP ≥ 85 mm Hg or Treatment of previously diagnosed hypertension

4.	Raised fasting plasma glucose (FPG) ≥ 100 mg/dL (5.6 mmol/L), or previously diagnosed type 2 diabetes

Participants were interviewed and examined clinically to get required information. Semi structured schedule was used to gather information regarding background characteristics and lifestyle related information. Socio-economic status was classified using Modified Kuppuswamy Socio-economic status scale [11]. Physical activity was assessed on the basis of occupation and accordingly it was classified in to sedentary, moderate and heavy type [12]. Anthropometric measurements which include waist circumference, weight, and height were done using standard methods. Body Mass Index (BMI) was classified according to WHO classification. Blood pressure measurements were done as per JNC VII guidelines [13]. Required biochemical parameters: triglycerides (TG), high density lipids (HDL) and fasting blood sugar (FBS) levels were estimated in participants who remained fasting for 12 hours prior to sample collection. The blood sample was collected using standard blood sample collection procedure. After labeling blood sample vials, they were transported to biochemistry laboratory of Government Medical College and Hospital Chandigarh for further processing.

Statistical tests like Chi square test, Normal tests of proportions, Student's t-test, and Analysis of variance (ANOVA) were applied. Kappa coefficient was used for studying agreement between IDF and ATP-III criteria [14]. SPSS-12 software was used for data analysis.

Ethical guidelines of Helsinki (1996) were followed. Informed consent was taken from all the participants and any risks involved during investigation were cared of medically to the possible extent. Information regarding MS was also provided to the participants prior to survey. Participants were imparted knowledge regarding prevention of MS after conducting the interview.

## Results

Out of 605 participants, 290 (47.9% were males) and 315 (52.1% were females). During sampling, proportional allocation was given to urban, rural and slum populations, as 481 (79.5%), 63 (10.4%), and 61 (10.1%) participants were from respective populations. Participants from all of the socioeconomic classes and religions were included in this study. Table 3 and Table 4 show the prevalence of MS and its association with various socio-demographic factors by IDF and ATP-III criteria respectively. According to IDF, 287 (47.4%) respondents were having MS and it was more in females 171 (54.3%) as compared to males 116 (40%). Prevalence estimates were less using ATP-III, as 233 (38.5%) individuals had MS. However, similar trends in favor of the female gender were seen, as 141 (44.8%) females compared to 116 (31.7%) males were having MS.

**Table 3:** Metabolic Syndrome (IDF) with various socio-demographic factors

Socio demographic factor	Metabolic Syndrome (IDF)		Total No. (% age)	P value
			
	Yes No. (% age)	No No.(% age)		
**Gender**				

**Male**	116 (40.4) (40.0)*	174 (54.7) (60.0)*	290 (47.9) (100.0)*	**p < .001**
	
**Female**	171 (59.6) **(54.3)**	144 (45.3) **(45.7)**	315 (52.1) **(100.0)**	

**Age**				

**18-25 yrs**	5 (1.7) **(9.4)**	48 (15.1) **(90.6)**	53 (8.8) **(100.0)**	**p < .001**
	
**26-35 yrs**	35 (12.2) **(30.7)**	79 (24.8) **(60.3)**	114 (18.8) **(100.0)**	
	
**36-49 yrs**	114 (39.7) **(50.7)**	111 (34.9) **(49.3)**	225 (37.2) **(100.0)**	
	
**50-59 yrs**	56 (19.5) **(61.5)**	35 (11.0) **(38.5)**	91 (15.0) **(100.0)**	
	
**60-65 yrs**	42 (14.6) **(63.6)**	24 (7.5) **(36.4)**	66 (10.9) **(100.0)**	
	
**>65 yrs**	35 (12.2) **(62.5)**	21 (6.6) **(37.5)**	56 (9.3) **(100.0)**	

**Mean ± SD**	**49.46 ± 13.24**	**44.96 ± 14.88**	**44.99 ± 14.74**	

**Background**				

**Urban**	249 (86.8) **(51.8)**	232 (73.0) **(48.2)**	481 (79.5) **(100.0)**	**p < .001**
	
**Rural**	26 (9.1) **(41.3)**	37 (11.6) **(58.7)**	63 (10.4) **(100.0)**	
	
**Slum**	12 (4.2) **(19.7)**	49 (15.4) **(80.3)**	61 (10.1) **(100.0)**	

**Socioeconomic status**				

**Upper-upper**	9 (3.1) **(64.3)**	5 (1.6) **(35.7)**	14 (8.8) **(100.0)**	**p = .006**
	
**Upper-middle**	190 (66.2) **(51.6)**	178 (56.0) **(48.4)**	368 (60.8) **(100.0)**	
	
**Upper-lower**	55 (19.2) **(44.4)**	69 (21.7) **(55.6)**	124 (20.5) **(100.0)**	

**Lower**	33 (11.5) **(33.3)**	66 (17.7) **(66.7)**	99 (16.4) **(100.0)**	

**Religion**				

**Hindu**	157 (54.7) **(42.2)**	215 (67.6) **(57.8)**	372 (61.5) **(100.0)**	**p = .002**

**Sikh**	121 (42.2) **(58.2)**	87 (27.4) **(41.8)**	208 (34.4) **(100.0)**	
	
**Muslim**	7 (2.4) **(35.0)**	13 (4.1) **(65.0)**	20 (3.3) **(100.0)**	
	
**Christian**	2 (0.7) **(40.0)**	3 (0.9) **(60.0)**	5 (0.8) **(100.0)**	

**Physical activity**				

**Sedentary**	259 (90.2) **(51.4)**	245 (77.0) **(48.6)**	504 (83.3) **(100.0)**	**p < .001**
	
**Moderate**	28 (9.8) **(30.1)**	65 (20.4) **(69.9)**	93 (15.4) **(100.0)**	
	
**Heavy**	0 (0.0) **(0.0)**	8 (2.5) **(100.0)**	8 (1.3) **(100.0)**	

**BMI**				

**Underweight**	0 (0.0) **(0.0)**	18 (5.7) **(100.0)**	18 (3.0) **(100.0)**	**p < .001**
	
**Normal**	51 (17.8) **(19.2)**	215 (67.6) **(80.8)**	266 (44.0) **(100.0)**	
	
**Pre-obese**	162 (56.4) **(67.5)**	78 (24.5) **(32.5)**	240 (39.7) **(100.0)**	
	
**Obese Class I**	63 (22.0) **(91.3)**	6 (1.9) **(8.7)**	69 (11.4) **(100.0)**	
	
**Obese Class II**	9 (3.1) **(90.0)**	1 (0.3) **(10.0)**	10 (1.7) **(100.0)**	
	
**Obese Class III**	2 (0.7) **(100.0)**	0 (0.0) **(0.0)**	2 (0.3) **(100.0)**	
	
**Mean ± SD**	**27.89 ±**	**23.16 ± 3.17**	**25.40 ± 4.13**	
	
**Total**	287 (100.0) **(47.4)**	318 (100.0) **(52.6)**	605 (100.0) **(100.0)**	

*Percentage in second row (in bold letters) represents percentage within that group in Table 3 and Table 4

**Table 4:** Metabolic Syndrome (ATP-III) with various socio-demographic factors

Socio demographic factor	Metabolic Syndrome (ATP-III)		Total No. (% age)	p value
			
	Yes No. (% age)	No No. (% age)		
**Gender**				

**Male**	116 (39.5) **(31.7)**	198 (53.2) **(68.3)**	290 (47.9) **(100.0)**	**p < .001**
	
**Female**	141 (44.8) **(44.8)**	174 (46.8) **(55.2)**	315 (52.1) **(100.0)**	

**Age**				

**18-25 yrs**	4 (1.7) **(7.5)**	49 (13.2) **(92.5)**	53 (8.8) **(100.0)**	**p < .001**
	
**26-35 yrs**	25 (10.7) **(21.9)**	89 (23.9) **(78.1)**	114 (18.8) **(100.0)**	
	
**36-49 yrs**	87 (37.3) **(38.7)**	138 (37.1) **(61.3)**	225 (37.2) **(100.0)**	
	
**50-59 yrs**	48 (20.6) **(52.7)**	43 (11.6) **(47.3)**	91 (15.0) **(100.0)**	
	
**60-65 yrs**	38 (16.3) **(57.6)**	28 (7.5) **(42.4)**	66 (10.9) **(100.0)**	
	
**>65 yrs**	31 (13.3) **(55.4)**	25 (6.7) **(44.6)**	56 (9.3) **(100.0)**	

**Mean ± SD**	**50.55 ± 13.24**	**41.51 ± 14.58**	**44.99 ± 14.74**	

**Background**				

**Urban**	198 (85.0) **(41.2)**	283 (76.1) **(58.8)**	481 (79.5) **(100.0)**	**p = .009**
	
**Rural**	22 (9.4) **(34.9)**	41 (11.0) **(65.1)**	63 (10.4) **(100.0)**	
	
**Slum**	13 (5.6) **(21.3)**	48(12.9) **(78.7)**	61 (10.1) **(100.0)**	

**Socioeconomic status**				

**Upper-upper**	9 (3.9) **(64.3)**	5 (1.3) **(35.7)**	14 (8.8) **(100.0)**	**p = .021**
	
**Upper-middle**	153 (65.7) **(41.6)**	215 (57.8) **(58.4)**	368 (60.8) **(100.0)**	
	
**Upper-lower**	38 (16.3) **(30.6)**	86 (23.1) **(55.6)**	124 (20.5) **(100.0)**	
	
**Lower**	33 (14.2) **(33.3)**	66 (17.7) **(66.7)**	99 (16.4) **(100.0)**	

**Religion**				

**Hindu**	131 (56.2) **(35.2)**	241 (64.8) **(64.8)**	372 (61.5) **(100.0)**	**p = .100**

**Sikh**	94 (40.3) **(45.2)**	114 (30.6) **(54.8)**	208 (34.4) **(100.0)**	
	
**Muslim**	6(2.6) **(30.0)**	14(3.8) **(70.0)**	20 (3.3) **(100.0)**	
	
**Christian**	2 (0.9) **(40.0)**	3 (0.8) **(60.0)**	5 (0.8) **(100.0)**	

**Physical activity**				

**Sedentary**	211 (90.6) **(41.9)**	293 (78.8) **(58.1)**	504 (83.3) **(100.0)**	**P < .001**
	
**Moderate**	22 (9.4) **(23.7)**	71 (19.1) **(76.3)**	93 (15.4) **(100.0)**	
	
**Heavy**	0 (0.0) **(0.0)**	8 (2.2) **(100.0)**	8 (1.3) **(100.0)**	
	
**BMI**				
	
**Underweight**	1 (0.4) **(5.6)**	17 (4.6) **(94.4)**	18 (3.0) **(100.0)**	**P < .001**
	
**Normal**	51 (21.9) **(19.2)**	215 (57.8%) **(80.8)**	266 (44.0) **(100.0)**	
	
**Pre-obese**	110 (47.2) **(45.8)**	130 (34.9) **(54.2)**	240 (39.7) **(100.0)**	
	
**Obese Class I**	60 (25.8) **(87.0)**	9 (2.4) **(13.0)**	69 (11.4) **(100.0)**	
	
**Obese Class II**	9 (3.9) **(90.0)**	1 (0.3) **(10.0)**	10 (1.7) **(100.0)**	
	
**Obese Class III**	2 (0.9) **(100.0)**	0 (0.0) **(0.0)**	2 (0.3) **(100.0)**	
	
**Mean ± SD**	27.95 ± 4.15	23.81 ± 3.23	**25.40 ± 4.13**	
	
**Total**	233 (100.0) **(38.5)**	372 (100.0) **(61.5)**	605 (100.0) **(100.0**	

Mean age of study population was 44.99 ± 14.74 yrs, but it was more in respondents with MS with both criteria. It was 49.46 ± 13.24 yrs in respondents with MS according to IDF (p < .001) and 50.55 ± 13.24 yrs in respondents with MS according to ATP-III (p < .001). Increasing trends of prevalence with age were seen with both IDF and ATP-III.

MS was more prevalent in urban area, by using both criteria. About 249 (51.8%) urban residents were having MS (IDF) as compared to 26 (41.3%) rural residents and 12 (19.7%) slum dwellers (p < .001). Similarly, prevalence of MS (ATP-III) was also more in urban residents 198 (41.2%) as compared to 22 (34.9%) rural residents and 13 (21.3%) slum dwellers (p = .009).

MS was more prevalent among respondents belonging to upper socioeconomic as compared to lower classes and there association was statistically significant (p = .006 and p = .021 respectively for IDF and ATP-III). MS was more prevalent among respondents belonging to Sikh religion. Prevalence was 58.2% in Sikhs followed by Hindus (42.2%), Christians (40%) and Muslims (35%) by IDF and it was statistically significant (p = .002). By ATP-III prevalence among Sikhs was 45.2%, Hindus (35.2%), Christians (40%), and Muslims (30%) but this association was statistically insignificant (p = .100).

It has been found that type of occupation, is significantly associated with MS (IDF) as 259 (51.4%) respondents involved in sedentary habitat were having MS as compared to 28 (30.1%) respondents of moderate type of work and none of the respondents involved in heavy work were having MS (p < .001). Parallel results were found by using ATP-III too. As 211 (41.9%) participants involved in sedentary habitat MS as compared 22 (23.7%) involved in moderate type of physical activity and none of the respondents involved in heavy activity had MS (p < .001).

Highly significant association of BMI with MS has been found by using both IDF and ATP-III criteria. None of the respondents from underweight group and only 51 (19.2%) individuals with normal BMI had MS (IDF). Prevalence of MS (IDF) increased from 67.5% in respondents from preo-obese class to 100% in respondents of obese class III. This association was highly significant (p < .001). Same trends were found by using ATP-III also (p < .001). Mean BMI of study population was low i.e. 25 ± 4.13 yrs as compared to the respondents with MS (IDF) 27.89 ± 3.02 years and respondents with MS (ATP-III) 27.95 ± 4.15 years.

The agreement between ATP-III and IDF criteria was found by using kappa coefficient of agreement and it was 0.636 and it is highly significant (P < .001).

## Discussion

### Prevalence world wide IDF vs. NCEP ATP-III

In the present study prevalence of MS was estimated by using both IDF and ATP-III criteria and overall prevalence rates were found as 47.5% and 38.5% respectively. Higher prevalence (by using IDF) can be explained by the lower cut-off points adopted by this new definition. In IDF, central obesity is the major criterion, its cut off is ethnic specific and is lower for Indians than used by original ATP-III. Another difference among two definitions is lower cutoff for FBS by IDF, which is > 100 as compared to > 110 in ATP-III.

The higher prevalence by new IDF definitions is comparable with other reports. Hu et al conducted a study in Finland and found that prevalence was 39.1% by ATP-III and 45.3% by IDF definition [15]. Similarly study done by Can et al concluded that prevalence was 42% by IDF and 38% by ATP-III in Turkish adults [16]. Similar results were also found by Harzallah et al [17] in Arab population (45.5% by IDF vs. 24.3% by ATP-III).

### Prevalence in India

In India very few studies had been done to find prevalence of MS and most of the studies which are available in literature had used ATP-III. But Deepa et al [18] compared the prevalence of MS in south Indian population by various definitions and found that by IDF 25.8% individuals > 20 yrs were having MS as compared to 18.3% by ATP-III. Another study from Bangalore by Kanjlal et al [19] concluded that prevalence of MS (by ATP-III) was 40.3% as compared to 34.9% by IDF definition. But no comparable study from north India is present in literature which has compared these two definitions. Gupta et al estimated the prevalence in Bhatia community in Rajasthan by ATP-III definition and it was 36.2% in males and 47.8% in females [20].

Reddy et al [21] conducted a multicentric study in subjects 20-69 yrs of age belonging to industrial employees or their family members. They used ATP-III and higher prevalence was found in Bangalore i.e. 38.8% comparable to current study (38.5%), Trivandrum 37.9%, Hyderabad 33.0%, Lucknow 29.0% but lower prevalence was found in Nagpur and Dibrugarh High prevalence of MS in present study could be attributed to modern life style adopted by residents of a highly urbanized population of Chandigarh, an Union Territory of India,

### MS with age and gender

In this study prevalence of MS (IDF) was highest i.e. 63.6% in age group of 60-65 years followed by age group of > 65 years i.e. 62.5% and lowest among age group of 18-25 years i.e. 9.4%. Prevalence was more among females than males by both IDF (M = 40.0%, F = 54.3%) and ATP-III (M = 31.7%, F = 44.8%). An overall increasing trend was reported in prevalence rates with increasing age by both definitions. This finding was in concordance with the study conducted by Kanjlal et al in Bangalore [19], who reported maximum prevalence in age group of 50-59 years and Reddy et al [21] who reported maximum prevalence among age group > 60 years in multi-centric industrial population of India. Similar trends were also reported by a study done by Taylor et al [22] in African-American population, who studied the prevalence in age group 21-94 years and maximum prevalence was seen in 65-74 year age group and lowest was seen among 21-34 year age group. Prevalence of MS was 44.8% in females and 33.4% among males. Another study done by Hildrum et al [23] in Norwegian population also found the similar trends in prevalence according to age and gender.

### Urban Rural Prevalence comparison

Prevalence of MS was highest among urban residents (51.8%, 41.2%) followed by rural population (41.3%, 34.9%) and prevalence in slum residents was lowest (19.7%, 21.3%) by both IDF and ATP-III definitions. But interestingly prevalence of MS by ATP-III was more among slum residents as compared to IDF definition. It could be due to less prevalence of central obesity among slum residents because of less sedentary lifestyle and central obesity is a major criterion in IDF as compared to ATP-III. Mahadik et al [24] also compared the prevalence among urban and rural populations of India and prevalence was more among urban as compared to rural residents (35.2% vs. 20.6%). Ramirez-Vargas et al [25] also found that prevalence of MS was more in urban as compared to rural populations (45.4% vs. 27.6%) in Oaxaca, Mexico. Another study by Sarkar et al [26] also revealed the effect of urbanization by comparing the prevalence of MS among two tribes of sub-Himalayan region of India.

### MS with Religion

Present study also showed the significant correlation between MS and religion. Sikh community had highest prevalence of MS (58.2%, 45.2%) followed Hindus (42.2%, 35.2%) by both IDF and ATP-III definitions but maximum number of respondents were from Hindu religion (61.5%). This difference occurred probably because of difference in the dietary habits of different communities. No comparable study could be found in the literature.

### MS with Socioeconomic Status

This study had shown the significant association of MS with socioeconomic status of respondents. MS was found to be more prevalent among subjects belonging to upper socioeconomic status as compared to lower classes. This finding was in concordance to the study done by Mohan et al [27] in South India where in significant difference in prevalence of MS was found according to socioeconomic status. MS was higher in middle compared to low income groups But in studies from developed countries and have shown the contrast results as Dallongeville et al [28] found that household income and education had an inverse relation with MS in France.

### Agreement of IDF and NCEP ATP-III

Significant agreement between ATP-III and IDF criteria for detecting MS indicates that we can use either of these two criteria for detecting MS. Optimum decisions may depend upon epidemiological situations. Choi et al [29], reported the agreement between ATP-III and the IDF as (κ = 0.54) in the Korean population.

## Conclusions

This study concludes that Metabolic Syndrome is highly prevalent in the urbanized community. Its contributory complications demand, screening of individuals in the community at the earliest, so that lifestyle modifications strategies may be adopted at an early age. Significant agreement between ATP-III and IDF criteria suggests possible use of IDF which restricts the blood investigations only if of central obesity is present as it is a major criterion. IDF may be a more feasible, practical and cost effective approach in the community set-up. This preliminary study in the region with IDF definition sets the background for future prospective studies, regarding causation, prevention and management of this syndrome.

## Limitations

In this study only demographic factors are presented, risk factor analysis should have been done by using prospective study.

## Competing interests

The authors declare that they have no competing interests.

## Authors' contributions

CM was responsible for the conception of the research question, collection, analysis and interpretation of data; and writing of the manuscript. NKG, MKS, NA and RS were responsible for conception of the research question, study design and coordination of research project. DKW was responsible for statistical analysis, interpretation of data and provided critical feedback to the manuscript for important intellectual content. JK participated in systematic biochemical analysis of the project. GS assisted in data collection, drafting of the manuscript and revised it critically for important intellectual content. All authors read and approved the final manuscript.
